# Advancing human ovarian biology in tandem with clinical care: considerations for collecting ovarian tissue for research after oophorectomy for tissue cryopreservation

**DOI:** 10.3389/fendo.2026.1866925

**Published:** 2026-06-03

**Authors:** Margaret A. Brunette, Karen Burns, Holly Hoefgen, Shuo Xiao, Francesca E. Duncan, Monica M. Laronda, Ariella Shikanov, Mary Zelinski, Veronica Gomez-Lobo

**Affiliations:** 1 Division of Pediatric and Adolescent Gynecology, Eunice Kennedy Shriver National Institute of Child Health and Human Development, Bethesda, MD, United States; 2 Cancer and Blood Diseases Institute, Cincinnati Children’s Hospital Medical Center, University of Cincinnati School of Medicine, Cincinnati, OH, United States; 3 St. Louis Children’s Hospital, Washington University School of Medicine in St. Louis, St. Louis, MO, United States; 4 Department of Pharmacology and Toxicology, Ernest Mario School of Pharmacy, Rutgers University, Piscataway, NJ, United States; 5 Environmental and Occupational Health Sciences Institute (EOHSI), Piscataway, NJ, United States; 6 Department of Obstetrics and Gynecology, Northwestern University Feinberg School of Medicine, Chicago, IL, United States; 7 Buck Institute for Research on Aging, Novato, CA, United States; 8 Stanley Manne Children’s Research Institute, Ann and Robert H. Lurie Children’s Hospital of Chicago, Chicago, IL, United States; 9 Department of Pediatrics, Northwestern University Feinberg School of Medicine, Chicago, IL, United States; 10 Department of Biomedical Engineering, University of Michigan, Ann Arbor, MI, United States; 11 Program in Cellular and Molecular Biology, University of Michigan, Ann Arbor, MI, United States; 12 Department of Obstetrics and Gynecology, University of Michigan, Ann Arbor, MI, United States; 13 Division of Reproductive and Developmental Sciences, Oregon National Primate Research Center, Beaverton, OR, United States; 14 Department of Obstetrics and Gynecology, Oregon Health & Science University, Portland, OR, United States

**Keywords:** human tissue, ovarian tissue cryopreservation (OTC), patient counselling, research samples, tissue processing

## Abstract

Increased access to OTC has enabled the collection, with patient consent, of research tissue samples from healthy premenopausal patients. These samples are a critical resource to advance our understanding of fundamental ovarian biology. When choosing a preservation method for the tissue collected for research purposes, it is important to define what outcomes are desired from tissue analysis. If tissue is preserved in certain ways the types of analyses available will be limited. This review details various processing methods that can be undertaken in the operating room, or after transportation, and examples of experimental approaches that are applicable to ovarian tissue research.

## Background

In 2019, the American Society for Reproductive Medicine deemed ovarian tissue cryopreservation (OTC) for fertility preservation no longer experimental (1). Increased access to OTC has enabled the collection, with patient consent, of research tissue samples from healthy premenopausal patients. These samples are a critical resource to advance our understanding of fundamental ovarian biology. Furthermore, the collection of these samples along with patient demographic data, hormone profiles, and menstrual/fertility history allows for a more nuanced analysis of the tissue. To date, this resource has provided unprecedented histological and molecular insight into the structure and function of healthy ovaries as well as those affected by conditions associated with reproductive dysfunction and premature ovarian insufficiency.

## Counseling and consent

Counseling regarding the use of ovarian tissue for research in the pediatric and adolescent/young adult (AYA) population can be included in the discussion of ovarian tissue cryopreservation (OTC) for fertility preservation. It may be helpful to provide a description of ongoing ovarian tissue research, either at the consenting institution or the institution that will ultimately receive the tissue.

Institutions that wish to collect ovarian tissue for research must have an IRB-approved protocol which allows for such a collection. The written consent should include explicit language regarding which portion of the ovary will be collected, what research can and cannot be performed with the tissue (e.g. creation of embryos) as well as who will have a right to use the tissue (possible future as yet unidentified investigators) and what, if any, personal identifiers will be shared. Informed consent should include the amount of tissue set aside for patient use and for research, with the stipulation that tissue for patient use is prioritized. The consent should also indicate whether any results from the research will be shared with the patients and if results will be deposited in any databases. Parents or guardians consent for those patients under 18; however patient assent must be obtained if the patient is of assenting age, which varies by institution. In the event that patients and parents disagree, further discussion with participants as well as consideration of an ethics consultation should be pursued. Patients over the age of 18 can provide their own consent.

## Removing a portion of the ovary

When removing a portion of ovarian tissue for research, different approaches have been undertaken. The original Oncofertility Consortium National Physician’s Cooperative protocol involved preserving 80% of the tissue for the patient’s own use and donation of up to 20% for research (2). Most sites collected either a biopsy or a small portion of cortex (less than 20%) after tissue processing due to concern about the clinical impact of removing a portion for research. When research tissue was collected post-processing, the pieces sent for research often did not include information regarding where on the ovary the sample was collected from. Furthermore, since the ovaries had already been largely de-cortified, some pieces sent for research did not actually include cortex.

**Figure 1:**
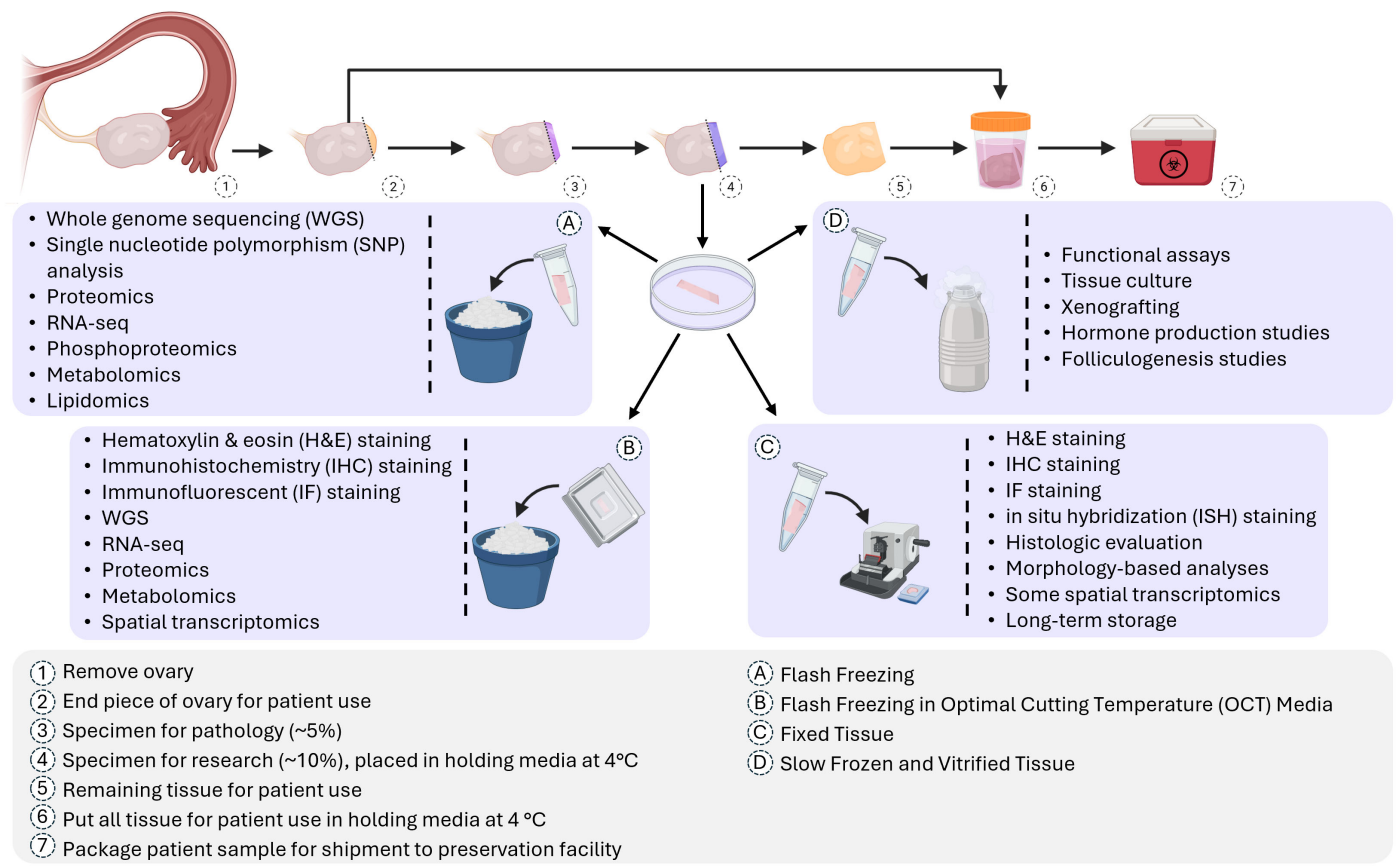
Tissue collection procedural overview. The schematic depicts the steps for tissue collection as performed at NIH, along with four research tissue processing approaches **(A–D)** and compatible experiment methodologies. Created with BioRender. Brunette, M. (2026) https://BioRender.com/dkxo9j8.

Although some sites initially performed small biopsies, more recently most sites have moved toward unilateral oophorectomy. This change should engender less concern regarding the effect of removing a small portion of the ovary for research (3). In addition, the region the research samples are taken from should be well documented, which will allow for more consistent evaluation of ovarian histology and function across different patients and studies (4). To collect tissue in a systematic manner, at NIH and Children’s National Hospital we perform a “salami cut” in the medial aspect of the lateral segment encompassing approximately 20% of the length of the long axis of the ovary under media in the operating room prior to sending tissue for processing (**Figure 1**). Care is taken to remove the ovary to be processed for the patient’s future use from the operating room prior to processing research tissue to avoid exposure to possible toxins such as fixative.

## Processing of research tissue

When choosing a preservation method for the tissue collected for research purposes, it is important to define what research outcomes are desired from tissue analysis. If tissue is preserved in certain ways (e.g. fixed or flash frozen), the types of analyses available will be limited. Here we detail various processing methods that can be undertaken in the operating room or after transportation at cold temperatures (5, 6). These preservation methods are presented separately, but they are not always mutually exclusive. For example, tissue preserved by OCT embedding, slow freezing, or vitrification can be fixed after sectioning or warming. In the following section, we discuss analysis methods and outputs in more detail.

### Flash freezing tissue

Flash freezing tissue stops all metabolic processes in the tissue immediately, making it particularly useful for “omics” research. This process is generally considered the gold standard for RNAseq/mRNAseq and phosphoproteomics. It is also well suited for whole genome sequencing (WGS), proteomics, metabolomics, lipidomics, epigenetic profiling, and single nucleotide polymorphism (SNP) analysis. The main caveat with this method is intracellular ice crystal formation. This makes it a poor choice for studies requiring tissue to be fixed for morphological analysis, such as hematoxylin & eosin staining (H&E), immunohistochemistry (IHC), and immunofluorescence staining (IF). Ice crystal formation should also be taken into consideration when tissue will be used for spatial analysis, as cell morphology is typically assessed along with spatial transcriptomics.

In an ideal situation, tissues would be immediately submerged in liquid nitrogen in the operating room, although that is not always possible. If liquid nitrogen is not available, the tissue can be placed in a container, such as a cassette or cryotube, and put it directly on dry ice until it can be moved to a liquid nitrogen dewar.

### Flash freezing in optimal cutting temperature media

Flash freezing in OCT media is ideal where frozen tissue needs to be cut into tissue sections. Key considerations for this method as compared to formalin-fixed paraffin embedded (FFPE) samples are: (1) unfixed sections can be produced, (2) block formation takes significantly less time, (3) it is considered gentler on the tissue, and (4) OCT media contains cryoprotectants to maintain cellular structure. However, it is difficult to cut OCT-embedded tissue into sections thinner than 8µm using the cryostat, while 2 – 5 µm sections can be cut from FFPE samples using a microtome. This tissue processing method is suitable for IHC, H&E, RNAseq, WGS, proteomics, metabolomics, and spatial transcriptomics.

In the operating room, the piece of tissue being preserved is placed in a Cryomold specimen holder filled with OCT media. The specimen holder is then placed in a “Seal’n Freeze Box” which allows for snap freezing using dry ice.

### Fixed tissue

Fixed, paraffin-embedded tissue is widely used to prepare tissue slides for analyses that rely heavily on cellular morphology (i.e. H&E, IHC, and spatial transcriptomics). A key consideration for fixing tissue is determining which fixative to use, as it can impact follicle morphology and appearance in histological analysis of the ovarian cortex (7). Tissue fixation, and subsequent paraffin-embedding, is also useful for long-term sample storage. The main drawback to tissue fixation is protein crosslinking, which may impact protein staining, although most IHC/IF protocols have an antigen retrieval step that helps negate some of the crosslinking. Conventionally, fixed tissues are considered unsuitable for “omics” work, but protocol adjustments can make paraffin embedded tissue usable with some “omics” and *in situ* hybridization platforms.

For this method, the tissue is placed in fixative in a specimen cup. However, if brought into the operating room, the container should not be opened until the tissue that is preserved for the patient’s use is sealed in a container with transport media and removed from the field. Once fixed, the tissue needs to be washed, stored, and paraffin embedded.

### Slow frozen and vitrified tissue

Slow freezing and vitrification are techniques widely used in OTC. There are many studies that compare the two methods head-to-head to evaluate outcomes. Although not all differences between methods are covered here, important factors include (1) the availability of slow freezing equipment, (2) the thickness of the tissue being cryopreserved, (3) protocol standardization, and (4) post-warming treatments (i.e. *in vitro* equilibration (8)). Regardless of cryopreservation method, pieces of cortex must be dissected. The size of those pieces should be carefully considered to balance cryoprotectant diffusion and reduce premature follicle activation by cutting into pieces that are too small (9).

There is overlap between possible analyses that can be conducted after tissue warming between the two methods (10). Slow freezing and vitrification are particularly useful when the analysis requires live cells, such as tissue culture and functional assays. The warming process and subsequent tissue handling are critical for obtaining optimal results, as both utilize cryoprotectants that can be toxic to the tissue if not effectively removed.

To collect tissue for slow freezing/vitrification, holding media is brought into the operating room for tissue transport. The tissue is then processed and preserved according to site-specific protocols (11).

## Methodologies and outcomes

As new methods and assays are developed, opportunities for analyzing this tissue continue to expand. Below is a list of approaches that are applicable to ovarian tissue research. This list is not exhaustive but can serve as a starting point to determine which data outputs are desired and thus which preservation methods should be used.

### Histological evaluation

Histological evaluation of ovarian tissue is often one of the first to be used to classify follicle stage and assess tissue health, and must be conducted using fixed tissue. As described above, maintaining cellular integrity is critical to classify the stages of individual follicles as they undergo folliculogenesis (12, 13). Basic H&E staining and imaging can be used to determine not only follicle density and stages, but also stromal cell density and give insight into tissue heterogeneity. The information collected from these images can be used in conjunction with methods and analyses discussed below to add context to other findings. This technique was used to quantify differences in follicle density and follicle stages across puberty in Tsui et al. (14).

### Visualization of molecular targets

IHC and IF both use antibodies to visualize targeted proteins, and the combination of protein location and cellular morphology can inform biological processes. DNA or RNA *in situ* hybridization (ISH) can be used to visualize molecular targets, sometimes in conjunction with protein staining (15). A limitation of these approaches is the number of targets that can be imaged in one tissue section, since each different molecule of interest must be visualized using either a different detection reagent or fluorophore.

### Omics approaches

Omics approaches can be subdivided into (1) bulk sequencing where the entire tissue is processed and sequenced as a single sample; (2) single-cell (sc) sequencing, where viable and intact cells are isolated and sequenced individually; and (3) single-nucleus (sn) sequencing, where nuclei are isolated and sequenced individually. The type of sequencing used depends on the scientific question. Bulk sequencing can give information about the tissue sample as a whole and is useful if information at the cellular level is not important to answer the research question. Single-cell sequencing can give information on specific cell types of interest and is useful to gather data about rare cells in a tissue sample. Single-nucleus approaches are useful when collecting intact cells is difficult. Importantly, data will not include molecules, such as mRNA, that have already entered the cytoplasm. The quantity of tissue available for processing also impacts the ability to collect data, as more tissue is needed for single-cell or single-nucleus analysis. For example, when processing samples for single-cell analysis, the tissue needs to be dissected and digested, which will likely yield fewer viable cells. For the specific case of human ovarian tissue, the amount of tissue needed can be particularly limiting due to the heterogeneity of this tissue (16). The process of dissection and digestion can also impact follicle activation and cellular processes, which can have implications for downstream analyses.

#### Genomics

Genomics methods are used to gain understanding of the DNA sequence present in samples. Sequencing approaches can be divided into whole genome sequencing (WGS), whole exome sequencing (WES), and targeted sequencing. WGS is used to find DNA variants, while WES focuses on the exome portion of the genome, with the exome being the protein-coding portion of the genome. These methods can find SNPs, insertions, deletions, and copy number variations (17). WGS/WES are commonly used for hypothesis generation. Targeted sequencing is focused on regions of interest in the genome. The benefit of targeted sequencing is that a smaller amount of data is collected, making computational analysis less cumbersome; however, the trade-off is that this method is hypothesis-driven rather than hypothesis-generating.

Publications regarding WGS and WES of healthy human ovarian tissue are sparse. This is likely because genomic testing can be done using cell samples that are more accessible (i.e. blood samples). In contrast, the literature includes WGS/WES studies of ovarian tumors using bulk sequencing methods, which is not surprising, since tumor genomes are often distinct (18–21). While genomic ovarian tissue sequencing is not usually necessary when studying normal ovarian function, obtaining genomic information using other cell samples may be beneficial for multi-omics studies.

#### Transcriptomics

The transcriptional activity of cells, where genetic information is read and converted to RNA, can give researchers insight into what genes are being expressed. When considering experimental approaches to evaluate mRNA for differential gene expression, snRNAseq is ideal for tissues with hard to dissociate cells (22). If the tissue is easily dissociated, scRNAseq has the added advantage of collecting data on all mRNA in each cell. For ovarian tissue specifically, it is important to consider that when dissociating cells for analysis in some platforms, large cells such as oocytes may be filtered out. The use of scRNAseq over snRNAseq may yield better data regarding protein synthesis since nuclear mRNA alone may be biased toward nuclear function mRNA (23). In addition to mRNA, it may be of interest to evaluate noncoding RNAs. In this case, different sequencing approaches can be taken for the evaluation of long noncoding (lnc), micro (mi), piwi-interacting (pi), and small nucleolar (sno) RNAs (24, 25).

A subset of transcriptomics approaches is spatial transcriptomics. This analysis integrates transcriptomic data with H&E or IF images. This technology is particularly powerful for ovarian tissue since follicle structure gives important context to the transcriptome. One drawback of this technology is that some platforms, such as Visium, do not have a resolution smaller than 30µm, capturing transcripts from adjacent cells within one data point (26). Importantly, newer technologies, such as Visium HD and Xenium, do allow for single-cell and subcellular resolution (27).

Transcriptomics has been gaining popularity in the ovarian tissue field and recent publications have investigated different cell types present in human ovarian tissue (28). Spatial transcriptomics has been used to study follicular atresia (29), ovarian aging (30), conditions associated with accelerated follicle atresia (31), and create a cellular atlas of ovarian tissue with spatial context (32).

#### Proteomics

Proteomics is similar to IHC and IF in the sense that they both provide protein information about a sample. It differs significantly from IHC and IF because proteomics characterizes many proteins present but the spatial context is lost. This methodology can be a powerful tool to evaluate posttranslational protein modifications and conduct protein abundance, phosphoproteomic, and glycoproteomic studies. Depending on the scientific question, this approach can be used to evaluate expression proteomics (i.e. disease state vs control state), structural proteomics (potential drug target identification), and functional proteomics (molecular mechanisms) (33, 34). As with transcriptomics, spatial proteomics platforms have been developed and can give important spatial context to proteomic data.

Proteomics research related to ovarian tissue has investigated the impact of gonadotoxic therapies (35), changes in expression during oogenesis and folliculogenesis (36), and spatial proteomics of the human ovary in a non-diseased state (37).

#### Metabolomics

Metabolomic profiling yields quantitative data about cellular processes and can indicate what metabolites are present at a tissue or organ level. Metabolic profiles also inform researchers how various lifestyles or disease states affect tissues and organs. The number of publications regarding ovarian metabolomics is not yet as high as other methodologies discussed. Lipidomics, a subfield of metabolomics, may be of particular interest in the ovarian biology field to better characterize lipid changes associated with PCOS (38, 39). Some published studies have evaluated differences in metabolites between different types of granulosa cells (40) and characterized metabolites in ovarian tumor tissues (41).

### Functional assays

These types of assays can be critical to characterize mechanisms of action and the impact of drug treatments. Functional assays are too varied and numerous to fully cover here; however, the critical aspect is that the tissue must maintain its native function. Xenografting of human tissue is the gold standard for functional analysis and allows for the evaluation of hormone production and folliculogenesis (42, 43).

## Summary

As the number of tissue analysis options increases and additional research is conducted using ovarian tissue, it is important that tissue be preserved and stored properly. Here we have detailed some key areas of analysis and the preservation methods best suited to different downstream applications and analyses, as summarized in **Figure 1**. Pre-planning experiments, when possible, is critical to ensure optimal and reproducible results.
